# Effects of acute and chronic disease on cell junctions in mouse liver

**DOI:** 10.17179/excli2022-5559

**Published:** 2023-01-02

**Authors:** Raf Van Campenhout, Bruno Cogliati, Mathieu Vinken

**Affiliations:** 1 Entity of In Vitro Toxicology and Dermato-Cosmetology, Department of Pharmaceutical and Pharmacological Sciences, Vrije Universiteit Brussel, Brussels, Belgium; 2 School of Veterinary Medicine and Animal Science, Department of Pathology, University of São Paulo, São Paulo, Brazil

**Keywords:** adherens junctions, tight junctions, gap junctions, liver, acute liver disease, chronic liver disease

## Abstract

Cell junctions, including anchoring, occluding and communicating junctions, play an indispensable role in tissue architecture and homeostasis. Consequently, malfunctioning of cell junctions is linked with a wide range of disorders, including in liver. The present study was set up to investigate the effects of acute and chronic disease induced by chemical compounds on hepatic cell junctions in mice. Mice were either overdosed with paracetamol or repeatedly administered carbon tetrachloride followed by sampling at 24 hours or 8 weeks, respectively. mRNA and protein expression levels of adherens, gap and tight junction components were measured in liver using reverse transcription quantitative real-time polymerase chain reaction analysis and immunoblot techniques, respectively. It was found that protein levels of the adherens junction building blocks β-catenin and γ-catenin, the gap junction components Cx26 and Cx32, and the tight junction constituent zonula occludens 2 were decreased, while mRNA levels of the adherens junction building block E-cadherin, and the tight junction constituent zonula occludens 2 and claudin 1 were upregulated following paracetamol overdosing. Repeated administration of carbon tetrachloride increased protein levels of E-cadherin, β-catenin, Cx26, Cx32, Cx43 and claudin 1. The latter was reflected at the mRNA level. In conclusion, acute and chronic liver disease have different effects on cell junctions in liver.

## Introduction

Cell junctions play an important role in maintaining tissue architecture and homeostasis (Garcia et al., 2017[8]; Adil et al., 2021[1]). These multiprotein complexes are embedded in the cell plasma membrane and can be classified according to their function, including anchoring, occluding and communicating junctions (Samiei et al., 2019[28]). Anchoring or adherens junctions establish adhesion between neighboring cells. They provide a mechanistic linkage and are built up by cytoplasmic cadherins and cytosolic catenins (Troyanovsky et al., 2021[31]). In this respect, the E-cadherin protein is seen as a guardian of liver physiology. E-cadherin, which is expressed by hepatocytes and biliary epithelial cells, is an important regulator of bile flow in the intrahepatic biliary network (Gonzalez-Sanchez et al., 2015[10]). β-catenin and γ-catenin are also produced by hepatocytes and maintain hepatocyte structure and function (Wickline et al., 2011[37]; Pradhan-Sundd et al., 2018[26]). β-catenin also participates in the canonical Wnt pathway, which controls the expression of genes involved in glutamine synthesis and biotransformation (Sekine et al., 2006[29]). Occluding junctions or tight junctions regulate cell polarity and form a permeability barrier to control the passage of ions and solutes into the paracellular space (Tsukita et al., 2019[32]; Krug and Fromm 2020[17]). Like adherens junctions, tight junctions are composed of transmembrane proteins and cytoplasmic proteins. Transmembrane components, including claudins, occludin, junctional adhesion molecules and the coxsackie-adenovirus receptor, provide the interaction between adjacent cells. These membrane-spanning proteins are tethered to cytoskeletal-proteins, such as zonula occludens, cingulin and symplekin proteins, to connect the cytoskeleton of neighboring cells (Shi et al., 2018[30]; Tsukita et al., 2019[32]; Lynn et al., 2020[21]). Functional tight junctions are required for normal liver functionality, in particular bile flow. Zonula occludens 2 and claudin 1 are indeed critical for the establishment of the blood-bile barrier and the control of paracellular permeability, respectively (Hadj-Rabia et al., 2004[12]; Grosse et al., 2012[11]; Itoh et al., 2021[15]; Xu et al., 2021[40]). Communicating or gap junctions are composed of 2 connexin (Cx) hemichannels of 2 adjacent cells, which in turn are built up by 6 Cx proteins. Gap junctions form water-filled tunnels that allow the diffusion of small and hydrophilic molecules between the cytosol of adjacent cells (Van Campenhout et al., 2022[33]). Cx26 and Cx32 are mainly expressed by hepatocytes, while most of the non-parenchymal liver cells harbor Cx43. Gap junctions, in particular Cx32-based channels, support liver-specific functions, including biotransformation, bile production, synthesis and secretion of proteins and control of carbohydrate metabolism (Van Campenhout et al., 2022[33]). Since adherens, tight and gap junctions are goalkeepers of hepatic homeostasis, it is not surprising that these structures are frequently implicated in liver disease (Garcia et al., 2017[8]; Adil et al., 2021[1]). The present study was set up in order to investigate the effects of acute and chronic liver disease on adherens, tight and gap junctions in liver. To mimic acute liver disease, mice were overdosed with paracetamol (PAR) for 24 hours, while chronic liver disease was simulated by repeated administration of carbon tetrachloride (CCL4) to mice for 8 weeks.

## Materials and Methods

### Animals and treatment

Male C57BL/6 mice (Jackson Laboratories, USA) were housed in the animal facility of the School of Veterinary Medicine and Animal Science of the University of São Paulo, Brazil. Animals were kept under controlled environmental conditions, in particular in a room with ventilation (16-18 air changes/ hour), relative humidity (45-65 %), controlled temperature (20-24 °C) and light/dark cycle 12:12, and were given water and balanced diet (Nuvital Nutrientes LTDA, Brazil) *ad libitum*. In the first set of experiments, mice were starved 15-16 hours *prior *to administration of PAR or 0.9 % sodium chloride (SAL) solution. PAR was dissolved in saline solution, slightly heated and injected (30-37 °C) intraperitoneally at 300 mg/kg body weight. After injection, mice regained free access to food. Mice were euthanized 24 hours following PAR overdosing by exsanguination during sampling under isoflurane-induced anesthesia. Another group of male C57BL/6 mice were administered corn oil (OIL) or CCL4 at a gradually increased dose for 8 weeks. Animals received 3 intraperitoneally injections weekly. The initial dose of CCL4 was 0.25 mg/kg body weight, and there were 0.25 mg weekly increments to the utmost dose of 1.25 mg/kg body weight. Mice were euthanized after completion of OIL or CCL4 treatment by exsanguination during sampling under isoflurane-induced anesthesia. Mouse blood, collected by cardiac puncture, was drawn into a heparinized syringe and centrifuged for 10 minutes at 1503×*g*, and serum was stored at -20 °C. Livers were snap-frozen in liquid nitrogen with storage at -80 °C. Both studies have been approved by the Committee on Bioethics of the School of Veterinary Medicine and Animal Science of the University of São Paulo, Brazil and all animals received humane care according to the criteria outlined in the “Guide for the Care and Use of Laboratory Animals”.

### Analysis of serum parameters

Alanine aminotransferase (ALT) (IU/L), aspartate aminotransferase (AST) (IU/L), alkaline phosphatase (ALP) (IU/L) and conjugated bilirubin (mg/dL) values were measured with an automated spectrophotometric Labmax 240 analyzer (Labtest Diagnostica, Brazil) or an automated bench-top dry chemistry analyzer (IDEXX Laboratories Ltd, UK) after appropriate dilution of collected serum samples. 

### Immunoblot analysis

Immunoblot analysis of liver tissue was performed as previously described with slight modifications (Willebrords et al., 2016[38]). In short, liver tissue was homogenized in radioimmunoprecipitation assay buffer containing Halt Protease Inhibitor Cocktail (Thermo Scientific, USA). Homogenates were centrifuged at 14000×*g* for 20 minutes at 4 °C. Protein concentrations in supernatants were determined using a commercial Pierce BCA Protein Assay kit (Thermo Scientific, USA). Protein lysates of 25 µg were resolved on sodium dodecyl sulphate polyacrylamide stain-free gels (Bio-Rad, USA) under reducing conditions. Separated proteins were transferred to nitrocellulose membranes (Bio-Rad, USA) and blocking was performed with 5 % milk in Tris-buffered saline solution containing 0.1 % Tween 20. Membranes were incubated overnight at 4 °C with primary antibody directed against E-cadherin, β-catenin, γ-catenin, Cx26, Cx32, Cx43, zonula occludens 2 and claudin 1 (Supplementary Table 1) followed by incubation for 1 hour at room temperature with a secondary HRP-conjugated goat anti-rabbit IgG antibody (1:1000) (Dako, USA). Detection of the proteins was carried out by means of enhanced chemiluminescence. For semi-quantification purposes, a normalization method based on total protein loading was used. Stain-free technology introduces the binding of a trihalo compound to tryptophan residues present in the sample. This allows visualization of total protein signals with low background and overcomes the drawbacks of housekeeping proteins (Diller et al., 2021[6]). All cell junction proteins were normalized against total protein loading and expressed as relative alterations compared to SAL-treated and OIL-treated animals for the acute and chronic liver disease model, respectively.

### Reverse transcription quantitative real-time polymerase chain reaction analysis

Reverse transcription quantitative real-time polymerase chain reaction (RT-qPCR) analysis of liver tissue was performed as explained elsewhere (Maes et al., 2016[24]). Briefly, total RNA was isolated from liver tissue using a GenElute Mammalian Total RNA Purification Miniprep Kit (Sigma, USA) and the On-Column DNase Digestion Set (Sigma, USA) according to the manufacturer's instructions. A NanoDrop ND-100 Spectrophotometer (Thermo Scientific, USA) was used to control purity and quantify RNA. Next, 2 µg RNA was reversely transcribed into cDNA with an iScript cDNA Synthesis Kit (Bio-Rad, USA) using an iCycler iQ (Bio-Rad, USA) followed by cDNA purification using a GenElute PCR Clean-Up Kit (Sigma, USA). cDNA products were quantitatively amplified using Taqman probes and primers specific for target and reference genes (Thermo Scientific, USA) (Supplementary Table 2). After running the samples in duplicate in a QuantStudio PCR System (Thermo Scientific, USA), the system's software was used to calculate quantification cycles. Stable reference genes for normalization were determined by geNorm using the qbase^+^ software (Biogazelle, Belgium) (Vandesompele et al., 2002[35]). The resulting ΔCq values of the test samples were normalized to those of the calibrator samples, yielding ΔΔCq values. Relative alterations (fold change) in RNA levels were calculated according to the 2^(-ΔΔCq)^ formula (Livak and Schmittgen, 2001[19]). Results were expressed as fold change of ΔΔCq values obtained from SAL-treated and OIL-treated mice.

### Statistical analysis

All data were expressed as mean ± standard deviation (SD). The number of biological replicates (*n)* and technical replicates (N) are specified for each analysis in the figure legends. Results were statistically processed by 2-tailed unpaired student t-tests and Welch's correction using GraphPad Prism7 software, with probability (*p*) values of less than or equal to 0.05 considered as significant.

## Results

### Effects of acute liver disease on hepatic cell junctions

**Figure 1:**
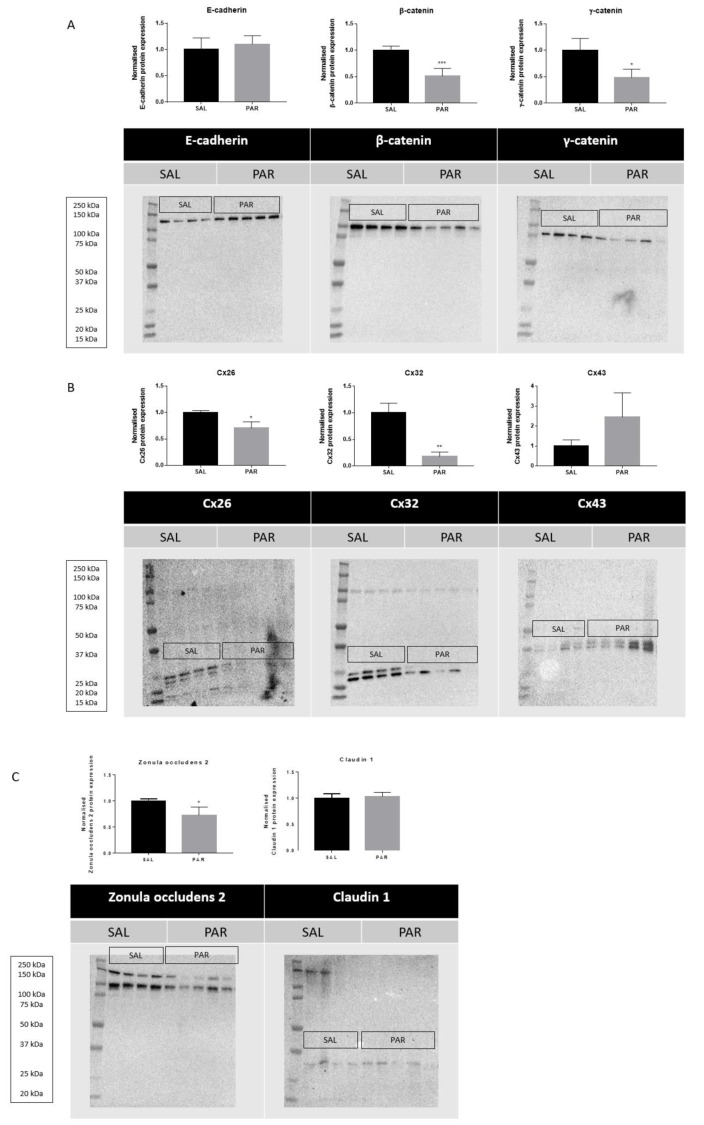
Effects of acute liver disease on protein levels of hepatic cell junction components. Mice were injected with SAL or PAR solution. After 24 hours, hepatic protein levels of E-cadherin, β-catenin, γ-catenin, Cx26, Cx32, Cx43, zonula occludens 2 and claudin 1 were assessed by immunoblot analysis, normalized against the total protein content and expressed as relative alteration compared to SAL-treated animals. Results were analyzed by 2-tailed unpaired student t-tests and Welch's correction. Data were expressed as means ± SD (*p ≤ 0.05; **p ≤ 0.01; ***p ≤ 0.001) (*n* = 4-5; N = 1).

**Figure 2:**
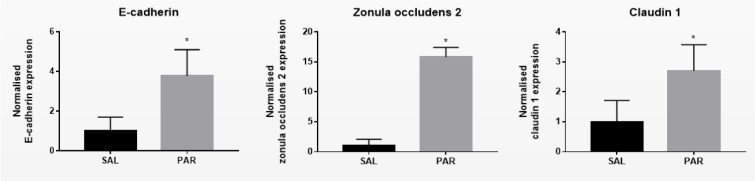
Effect of acute liver disease on mRNA levels of hepatic cell junction components. Mice were injected with SAL or PAR solution. After 24 hours, mRNA was extracted from the liver and subjected to RT-qPCR analysis of E-cadherin, β-catenin, γ-catenin, Cx26, Cx32, Cx43, zonula occludens 2 and claudin 1. Fold changes in RNA levels were calculated, where the average expression of SAL-treated animals was set to 1. Results were analyzed by 2-tailed unpaired student t-tests and Welch's correction. Data were expressed as means ± SD (*p ≤ 0.05) (*n* = 4-5; N = 2).

Overdosing mice with the analgesic drug PAR results in excessive accumulation of *N*-acetyl-p-benzoquinone imine (NAPQI). This triggers the formation of NAPQI-protein adducts, which in turn leads to mitochondrial dysfunction and nuclear DNA damage. Consequently, an overdose of PAR causes massive necrotic hepatic cell death and inflammation within 24 hours (Maes et al., 2016[23]). Measuring serum ALT levels is still considered as the most relevant clinical biomarker to assess hepatocellular injury, including PAR-evoked insults (Maes et al., 2016[23]). PAR overdosing indeed increased serum levels of ALT and AST after 24 hours (^***^p ≤ 0.001) (Supplementary Figure 1). Protein and mRNA levels of E-cadherin, β-catenin, γ-catenin, Cx26, Cx32, Cx43, zonula occludens 2 and claudin 1 were measured by immunoblot and RT-qPCR analysis, respectively. Whereas protein levels of β-catenin (^***^p ≤ 0.001) and γ-catenin (^*^p ≤ 0.05) (Figure 1A(Fig. 1)) decreased, mRNA quantities of E-cadherin increased (^*^p ≤ 0.05) (Figure 2(Fig. 2)). Hepatic protein expression levels of Cx26 (^*^p ≤ 0.05) and Cx32 (^**^p ≤ 0.01) (Figure 1B(Fig. 1)) were negatively affected. The effect of PAR overdosing was not mirrored at transcriptional level as no shifts in mRNA levels of Cx26, Cx32 and Cx43 were observed (Supplementary Figure 2). While liver protein levels of zonula occludens 2 (^*^p ≤ 0.05) (Figure 1C(Fig. 1)) declined, mRNA levels of zonula occludens 2 (^*^p ≤ 0.05) and claudin 1 (^*^p ≤ 0.05) (Figure 2(Fig. 2)) increased following PAR overdosing. Protein levels of E-cadherin, Cx43 and claudin 1 were unaffected upon PAR overdosing (Figure 1A-C(Fig. 1)).

### Effects of chronic liver disease on hepatic cell junctions

**Figure 3:**
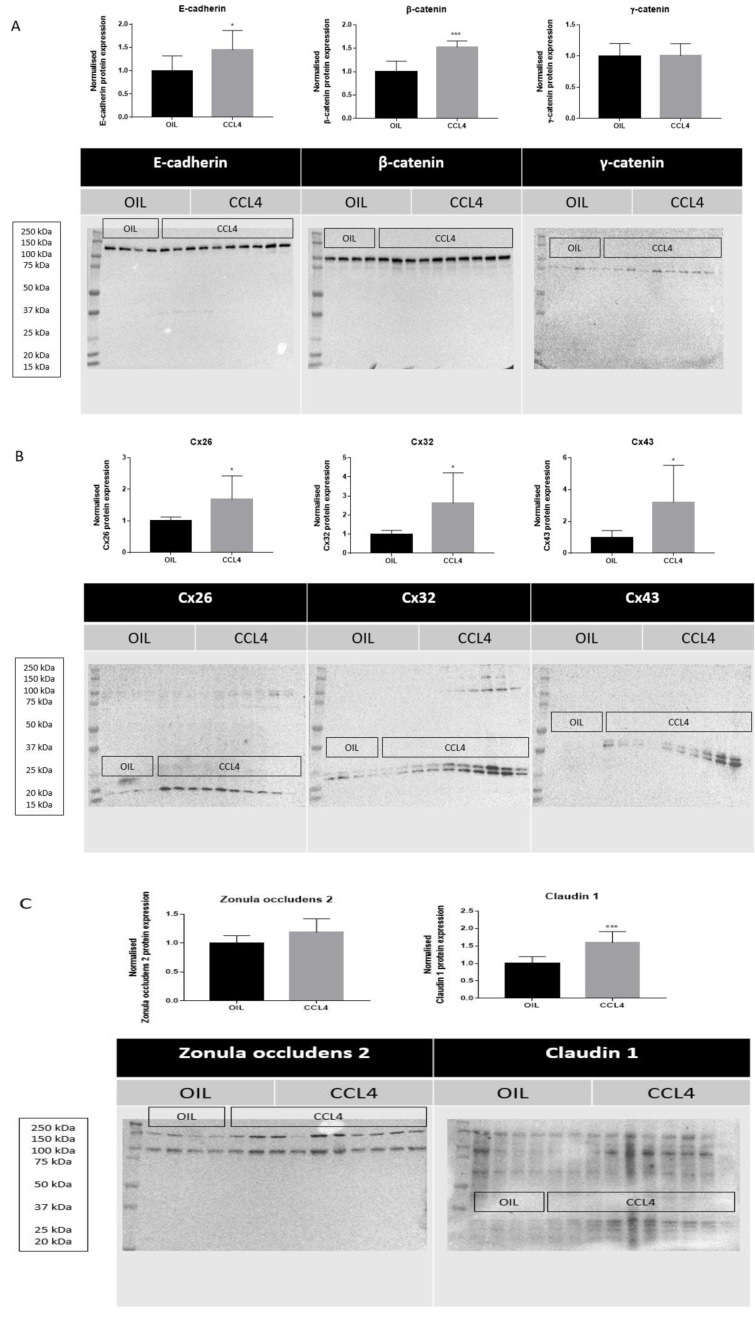
Effects of chronic liver disease on protein levels of hepatic cell junction components. Mice received injections with OIL or CCL4 for 8 weeks. Hepatic protein levels of E-cadherin, β-catenin, γ-catenin, Cx26, Cx32, Cx43, zonula occludens 2 and claudin 1 were assessed by immunoblot analysis, normalized against the total protein content and expressed as relative alteration compared to OIL-treated animals. Results were analyzed by 2-tailed unpaired student t-tests and Welch's correction. Data were expressed as means ± SD (*p ≤ 0.05; ***p ≤ 0.001) (*n* = 8-10; N = 1).

**Figure 4:**
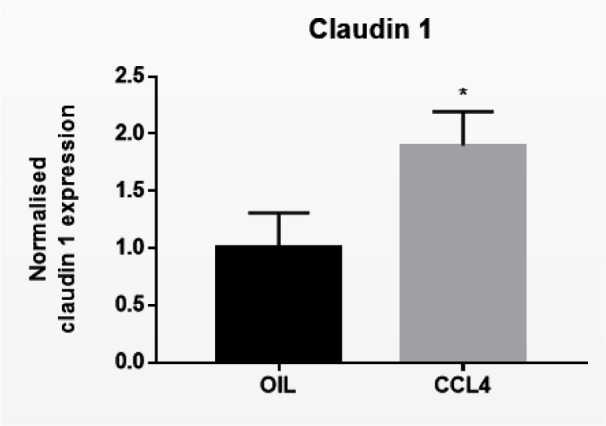
Effect of chronic liver disease on mRNA levels of hepatic cell junction components. Mice received injections with OIL or CCL4 for 8 weeks. mRNA was extracted from the liver and subjected to RT-qPCR analysis of E-cadherin, β-catenin, γ-catenin, Cx26, Cx32, Cx43, zonula occludens 2 and claudin 1. Fold changes in mRNA levels were calculated, where the average expression of OIL-treated animals was set to 1. Results were analyzed by 2-tailed unpaired student t-tests and Welch's correction. Data were expressed as means ± SD (*p ≤ 0.05) (*n* = 8-10; N = 2).

Following biotransformation of CCL4, the noxious trichloromethyl radical is generated in the liver. Trichloromethyl radical triggers various reactions involving free radicals and lipid peroxidation, which burgeons into the onset of cell death, inflammation and liver fibrosis (Yanguas et al., 2016[42]). CCL4 is widely used to trigger liver fibrosis in mice and repeated administration of CCL4 evokes chronic liver disease between 2 and 6 months (Ghallab et al., 2019[9]). Induction of chronic liver disease was evidenced by increased serum levels of ALT (^****^p ≤ 0.0001), AST (^***^p ≤ 0.001), total bilirubin (^*^p ≤ 0.05), and conjugated bilirubin (^****^p ≤ 0.0001) after 8 weeks (Supplementary Figure 3). Protein expression levels of E-cadherin (^*^p ≤ 0.05) and β-catenin (^***^p ≤ 0.001) (Figure 3A(Fig. 3)) were increased in mouse liver tissue following repeated CCL4 treatment, yet without changes in mRNA profiles (Supplementary Figure 4). mRNA (Supplementary Figure 4) and protein levels (Figure 3A(Fig. 3)) of γ-catenin were not altered upon CCL4 treatment. Hepatic Cx26 (^*^p ≤ 0.05), Cx32 (^*^p ≤ 0.05) and Cx43 (^*^p ≤ 0.05) (Figure 3B(Fig. 3)) protein levels were increased. Here again, these changes were not reflected at the transcriptional level (Supplementary Figure 4). Protein levels (^***^p ≤ 0.001) (Figure 3C(Fig. 3)) and mRNA quantities of claudin 1 (^*^p ≤ 0.05) (Figure 4(Fig. 4)) were positively affected upon CCL4 treatment. No shifts in mRNA (Supplementary Figure 4) and protein levels (Figure 3C(Fig. 3)) of zonula occludens 2 were observed.

## Discussion

The present study was set up in order to investigate the effects of acute and chronic liver experimentally induced in mice by exposure to chemical compounds on the expression of hepatic cell junction components. Given that anchoring, occluding and communicating junctions support liver homeostasis, hepatic cell junction components are often targeted in liver disease. The acute liver disease model used in this study relied on PAR overdosing and caused a reduced expression of β-catenin, γ-catenin, Cx26, Cx32 and zonula occludens 2 proteins in mouse liver. *In vitro *and* in vivo *studies previously demonstrated the malfunctioning of liver cell junctions upon PAR intoxication (Newsome et al., 2004[25]; Maes et al., 2016[22]; Gamal et al., 2017[7]). Likewise, PAR evoked disruption of tight junctions accompanied by a dose-dependent loss of zonula occludens 1 in cultures of human hepatoma cells and primary mouse hepatocytes (Gamal et al., 2017[7]). Furthermore, human hepatoma cells and primary human hepatocytes lost their adhesive capacity when exposed to serum of patients with PAR induced acute liver failure (Newsome et al., 2004[25]). Overdosing mice with PAR also deteriorated gap junction activity and decreased Cx32 protein levels (Maes et al., 2016[22]). Given that mRNA levels of E-cadherin, zonula occludens 2 and claudin 1 were upregulated in the present study, the effects of PAR on hepatic cell junction proteins seemed not be mediated by the transcriptional machinery. Malfunctioning of cell junctions and loss of hepatic cell junction components is associated with several other liver diseases. In this respect, downregulation of the expression of tight junction components is frequently linked with chronic biliary disease (Roehlen et al., 2020[27]). Similarly, the production of zonula occludens 1, occludin and claudin 1, is negatively affected in liver fibrosis (Wang et al., 2021[36]). mRNA and protein expression levels of most of these cell junction components are also decreased in cholestasis (Huang et al., 2021[14]). Furthermore, liver injury leads to dysfunction of gap junctions accompanied with changes in Cx26, Cx32 and Cx43 quantities (Yang et al., 2019[41]; Van Campenhout et al., 2022[33]). Accordingly, hepatic Cx32 expression is low in patients with non-alcoholic steatohepatitis and negatively correlates with increasing liver injury (Luther et al., 2018[20]). On the other hand, hepatitis C virus infection and hepatocellular carcinoma can cause the upregulation of hepatic tight junction protein expression (Roehlen et al., 2020[27]). Bile duct ligation-induced liver injury drastically increased adherens junction component quantities in mouse liver, which might be caused by hepatic repair processes (Li et al., 2007[18]; Clerbaux et al., 2019[3]; Van Campenhout et al., 2019[34]). Repeated administration of CCL4 was used to initiate chronic liver disease in mice in the present study. Hepatic protein levels of E-cadherin, β-catenin, Cx26, Cx32, Cx43 and claudin 1 were elevated in CCL4-treated mice. This might be related to type 2 epithelial to mesenchymal transition (EMT). Despite some controversy, type 2 EMT appears to be involved in liver fibrosis (Choi and Diehl, 2009[2]; Xie and Diehl, 2013[39]; Zhao et al., 2016[43]). During EMT, epithelial cells acquire a migratory character, start to express mesenchymal markers, like β-catenin, and evolve into collagen producing fibroblasts (Zhao et al., 2016[43]). Cells undergoing EMT do not only produce mesenchymal markers, but they also secure their epithelial nature by expressing epithelial-specific E-cadherin (Kalluri and Weinberg, 2011[16]). In this regard, advanced type 2 EMT activity might underly the increased expression of E-cadherin and β-catenin. In accordance with the surge in claudin 1 at the transcriptional and translational level, CCL4 has been reported to manifest an increase in hepatic tight junction components contributing to hepatic sinusoidal resistance and portal hypertension (Hintermann et al., 2016[13]). While there was no difference in Cx26, Cx32 and Cx43 mRNA expression in fibrotic mice, chronic liver disease induced expression of the corresponding proteins. Genetic ablation of Cx32 proteins revealed a role for Cx32 in tissue protection during liver fibrosis. Enhancement of Cx32 production may counteract hepatocyte damage and protect against chronic liver injury (Cogliati et al., 2016[5]). In addition, it has been reported that induced expression of hepatic Cx43 proteins in fibrotic mice has a function in the control and regulation of fibrogenesis. Cx43-deficient mice developed excessive liver fibrosis after repeated administration of CCL4 (Cogliati et al., 2011[4]). As a general conclusion, the present study showed that acute and chronic liver disease have different effects on cell junctions in liver.

## Declaration

### Acknowledgments

The authors wish to thank Miss Fien Haenen and Mr. Arne Loosen for their dedicated technical assistance. This work was financially supported by the Methusalem program of the Flemish Government and the University Hospital of the Vrije Universiteit Brussel-Belgium (Scientific Fund Willy Gepts).

### Conflict of interest

The authors declare that the research was conducted in the absence of any commercial or financial relationships that could be construed as a potential conflict of interest. 

## Supplementary Material

Supplementary information
