# Supplementary material for: Effects of acute and chronic disease on cell junctions in mouse liver
Source: EXCLI J. 2023 Jan 2;22:1–11. doi: 10.17179/excli2022-5559 (PMC9837383; doi:10.17179/excli2022-5559)
Supplement: Supplementary information [file EXCLI-22-1-s-001.pdf]

EXCLI Journal 2023;22:1-11 – ISSN 1611-2156
Received: October 28, 2022, accepted: November 15, 2022, published: January 02, 2023

Supplementary information to:

Original article:

EFFECTS OF ACUTE AND CHRONIC DISEASE ON CELL JUNCTIONS IN MOUSE LIVER

Raf Van Campenhout[1], Bruno Cogliati[2], Mathieu Vinken[1*]

[1] Entity of In Vitro Toxicology and Dermato-Cosmetology, Department of Pharmaceutical and Pharmacological Sciences, Vrije Universiteit Brussel, Brussels, Belgium
[2] School of Veterinary Medicine and Animal Science, Department of Pathology, University of São Paulo, São Paulo, Brazil

* **Corresponding author:** Prof. Mathieu Vinken, Ph.D., Pharm.D., E.R.T., Vrije Universiteit Brussel, Entity of In Vitro Toxicology and Dermato-Cosmetology, Laarbeeklaan 103, B-1090 Brussels, Belgium; Tel: +32-2-4774587; Fax: +32-2-4774582; E-mail: mathieu.vinken@vub.be

https://dx.doi.org/10.17179/excli2022-5559

This is an Open Access article distributed under the terms of the Creative Commons Attribution License (http://creativecommons.org/licenses/by/4.0/).

EXCLI Journal 2023;22:1-11 – ISSN 1611-2156
Received: October 28, 2022, accepted: November 15, 2022, published: January 02, 2023

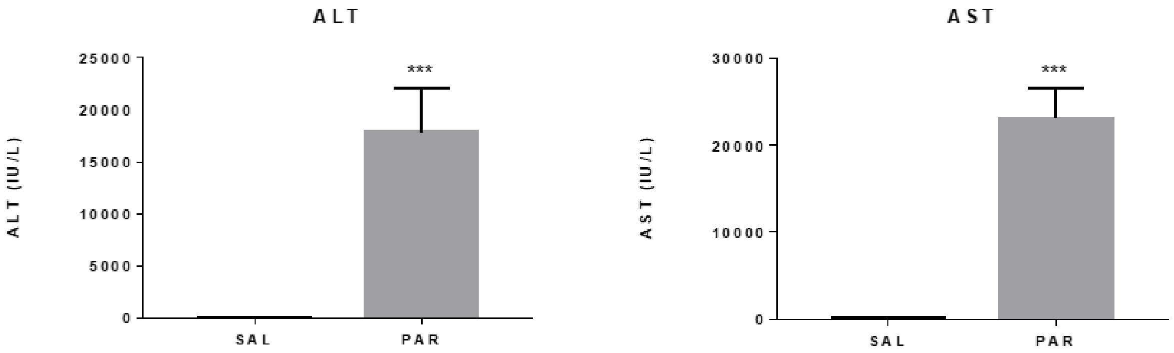

Supplementary Figure 1: Effects of acute liver disease on serum levels of ALT and AST. Mice were injected with SAL or PAR solution. After 24 hours, serum levels of ALT and AST were measured. Results were analyzed by 2-tailed unpaired student t-tests and Welch's correction. Data were expressed as means ± SD (****p* ≤ 0.001) (*n* = 4-5; N = 1).

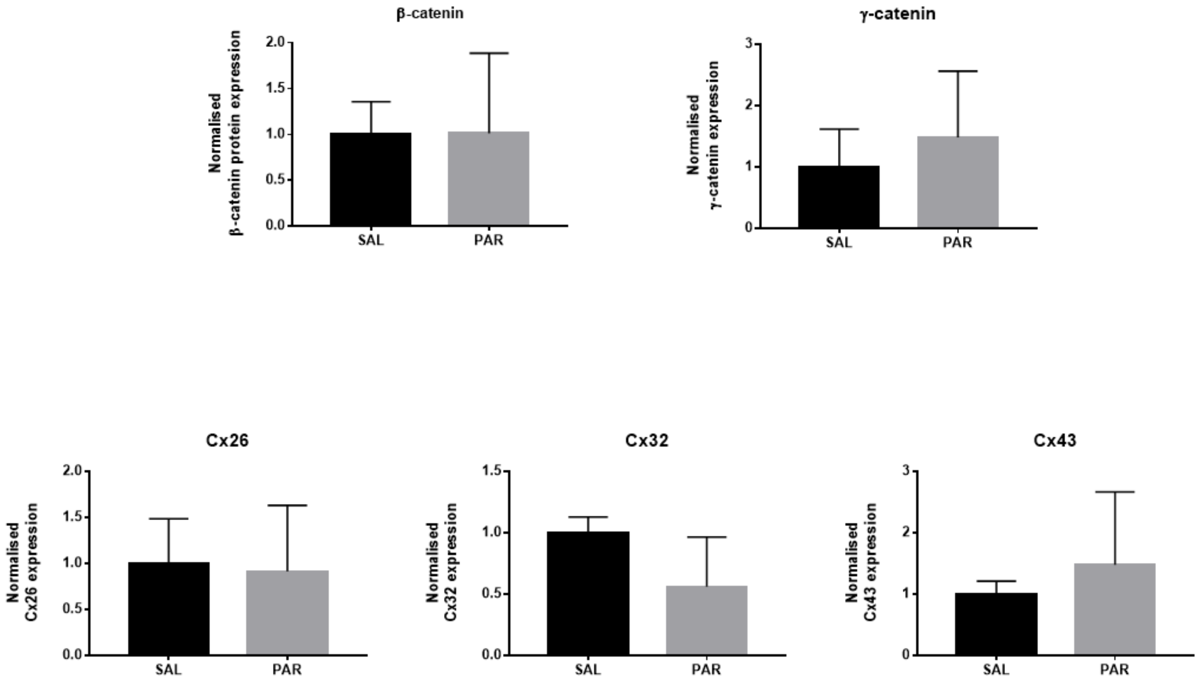

Supplementary Figure 2: Effect of acute liver disease on mRNA levels of hepatic cell junction components. Mice were injected with SAL or PAR solution. After 24 hours, mRNA was extracted from the liver and subjected to RT-qPCR analysis of E-cadherin, β-catenin, γ-catenin, Cx26, Cx32, Cx43, zonula occludens 2 and claudin 1. Fold changes in RNA levels were calculated, where the average expression of SAL-treated animals was set to 1. Results were analyzed by 2-tailed unpaired student t-tests and Welch's correction. Data were expressed as means ± SD (*n* = 4-5; N = 2).

EXCLI Journal 2023;22:1-11 – ISSN 1611-2156
Received: October 28, 2022, accepted: November 15, 2022, published: January 02, 2023

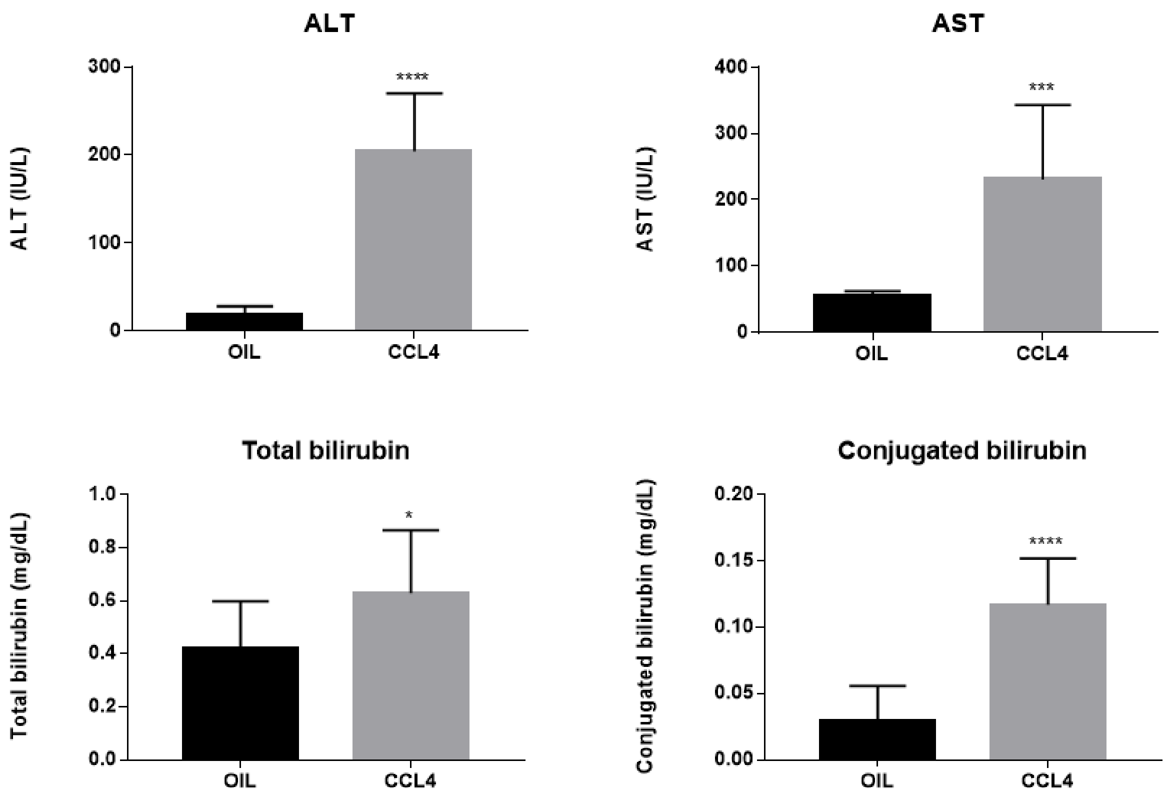

Supplementary Figure 3: Effects of chronic liver disease on serum levels of ALT, AST, total bilirubin and conjugated bilirubin. Mice were injected with OIL or CCL4 for 8 weeks. Serum levels of ALT, AST, total bilirubin and conjugated bilirubin were measured. Results were analyzed by 2-tailed unpaired student t-tests and Welch's correction. Data were expressed as means ± SD (*$p \le 0.05$; ***$p \le 0.001$; ****$p \le 0.0001$) (n = 8-10; N = 1).

EXCLI Journal 2023;22:1-11 – ISSN 1611-2156
Received: October 28, 2022, accepted: November 15, 2022, published: January 02, 2023

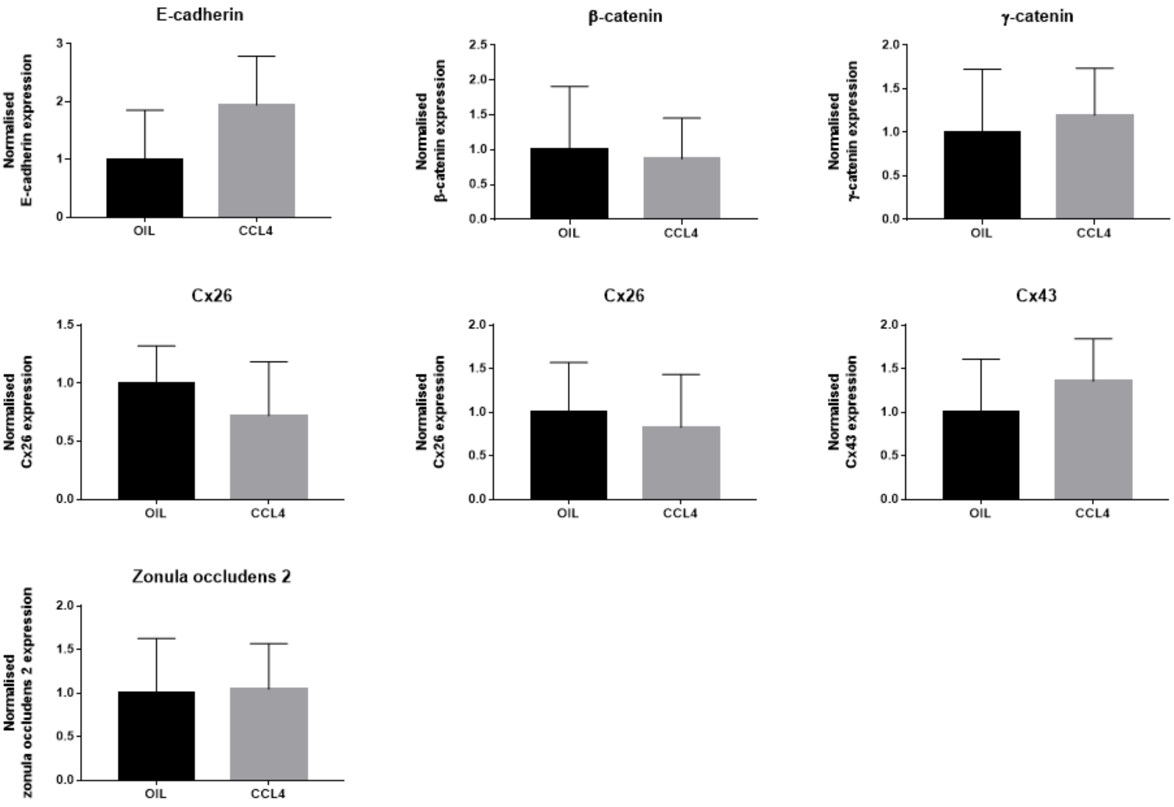

Supplementary Figure 4: Effect of chronic liver disease on mRNA levels of hepatic cell junction components. Mice received injections with OIL or CCL4 for 8 weeks. mRNA was extracted from the liver and subjected to RT-qPCR analysis of E-cadherin, β-catenin, γ-catenin, Cx26, Cx32, Cx43, zonula occludens 2 and claudin 1. Fold changes in mRNA levels were calculated, where the average expression of OIL-treated animals was set to 1. Results were analyzed by 2-tailed unpaired student t-tests and Welch's correction. Data were expressed as means ± SD (*n* = 8-10; N = 2).

EXCLI Journal 2023;22:1-11 – ISSN 1611-2156
Received: October 28, 2022, accepted: November 15, 2022, published: January 02, 2023

Supplementary Table 1: Primary antibodies used for immunoblot analysis

Antigen	Supplier	Cat. No.	Species	Type	Dilution
E-cadherin	Cell Signaling Technology	CST 3195	Rabbit	Monoclonal	1/1000
β-catenin	Abcam	Ab2365	Rabbit	Polyclonal	1/1000
γ-catenin	Cell Signaling Technology	CST 2309	Rabbit	Polyclonal	1/1000
Cx26	Thermo Scientific	51-2800	Rabbit	Polyclonal	1/1000
Cx32	Sigma	C3470	Rabbit	Polyclonal	1/1000
Cx43	Sigma	C6219	Rabbit	Polyclonal	1/1000
Zonula occludens 2	Cell Signaling Technology	CST 2847	Rabbit	Polyclonal	1/1000
Claudin 1	Cell Signaling Technology	CST 13255	Rabbit	Monoclonal	1/1000

Supplementary Table 2: Primers and probes for RT-qPCR analysis. Assay identification (ID), accession number, assay location, amplicon length and exon boundary of target and candidate reference genes are presented. (*Actb*, β-actin; *B2m*, β-2-microglobulin; *Cdh1*, E-cadherin, *Cldn1*, Claudin 1; *Ctnnb1*, β-catenin; *Gapdh*, Glyceraldehyde 3-phosphate; *Gja1*, Cx43; *Gjb1*, Cx32; *Gjb2*, Cx26; *Hhmbs*, Hydroxymethylbilane synthase; *Jup*, γ-catenin; *Tjp2*, Zonula occludens 2; *Ubc*, Ubiquitin C)

Gene Symbol	Assay ID	Accession number	Assay location	Amplicon size (base pairs)	Exon boundary
Actb (β-actin)	Mm00607939_s1	NM_007393.3	1233	115	6-6
B2m (β-2-microglobulin)	Mm00437762_m1	NM_009735.3	111	77	1-2
Cdh1 (E-cadherin)	Mm01247357_m1	NM_009864.2	1452	71	9-10
Cldn1 (Claudin 1)	Mm01342184_m1	NM_016674.4	442	76	1-2
Ctnnb1 (β-catenin)	Mm00483039_m1	NM_007614.3	2366	77	13-14
Gapdh (glyceraldehyde 3-phosphate)	Mm99999915_g1	NM_008084.3	265	107	2-3
Gja1 (Cx43)	Mm01179639_s1	NM_010288.3	2937	168	2-2
Gjb1 (Cx32)	Mm01950058_s1	NM_008124.2	466	65	1-1
Gjb2 (Cx26)	Mm00433643_s1	NM_008125.3	603	72	2-2
Hmbs (hydroxymethylbilane synthase)	Mm01143545_m1	NM_013551.2	473	81	6-7
Jup (γ-catenin)	Mm00550256_m1	NM_034723.1	1657	66	8-9
Tjp2 (Zonula occludens 2)	Mm00495620_m1	NM_011597.4	230	113	2-3
Ubc (Ubiquitin C)	Mm02525934_g1	NM_019639.4	370	176	2-2
